# 高效液相色谱-串联质谱法测定野生蘑菇中鹅膏肽类与色胺类毒素

**DOI:** 10.3724/SP.J.1123.2023.07013

**Published:** 2023-11-08

**Authors:** Leiqi LIU, Jingze CHEN, Wusheng FU, Cuiying TANG

**Affiliations:** 1.福建医科大学公共卫生学院, 福建 福州 350122; 1. School of Public Health, Fujian Medical University, Fuzhou 350122, China; 2.福建省人兽共患病研究重点实验室, 福建省疾病预防控制中心, 福建 福州 350012; 2. Fujian Provincial Key Laboratory for Zoonosis Research, Fujian Center for Disease Control and Prevention, Fuzhou 350012, China; 3.福建中医药大学药学院, 福建 福州 350122; 3. School of Pharmacy, Fujian University of Traditional Chinese Medicine, Fuzhou 350122, China; 4.福建农林大学食品科学学院, 福建 福州 350002; 4. Food Science College of Fujian Agriculture and Forestry University, Fuzhou 350002, China

**Keywords:** 高效液相色谱-串联质谱法, 鹅膏毒肽, 鬼笔毒肽, 脱磷裸盖菇素, 蟾蜍色胺, 蘑菇, high performance liquid chromatography-tandem mass spectrometry (HPLC-MS/MS), amanitins, phallotoxins, psilocin, bufotenine, mushroom

## Abstract

蘑菇毒素种类繁多,化学结构差异大,为了实现蘑菇中毒素的准确、高通量分析,本文采用固相萃取净化技术,以高效液相色谱-串联质谱法(HPLC-MS/MS)为分析手段,建立了蘑菇中5种鹅膏肽类、2种色胺类蘑菇毒素的分析方法。优化了色谱条件、质谱参数和样品提取净化方法,蘑菇干粉以含0.3%甲酸的甲醇提取,强阳离子交换柱(SCX)净化后,待测样液用T3色谱柱分离,以含0.1%甲酸的5 mmol/L乙酸铵水溶液、乙腈为流动相进行梯度洗脱,采用多反应监测模式(MRM)扫描,以基质匹配标准曲线法对鹅膏肽毒素定量,以同位素内标法对色胺类毒素定量。结果显示,7种毒素在一定的浓度范围内与峰面积(或峰面积比)均呈良好的线性关系(*r*^2^>0.99)。蟾蜍色胺、脱磷裸盖菇素、鹅膏肽类毒素的检出限(LOD)分别为2.0、5.0、10 μg/kg,定量限(LOQ)分别为5.0、10、20 μg/kg;以香菇干粉为基质,在3个水平下加标,5种鹅膏肽类毒素的回收率为71.8%~115%,相对标准偏差(RSD)为2.14%~9.92%; 2种色胺类毒素的回收率为80.6%~117%, RSD为1.73%~5.98%;与国家市场监管总局食品补充检验方法BJS 202008进行比对,结果表明鹅膏毒素含量具有可比性,无显著性差异(*p*>0.05)。该方法简便、快速,准确度和精密度较高,符合要求,适用于野生蘑菇中7种蘑菇毒素的检测。采用该法开展了福建省野生蘑菇中蘑菇毒素分布情况的调查,在全省9个设区市采集了59份野生蘑菇样品,采用核糖体DNA内转录间隔区(rDNA-ITS)分子条形码技术进行了种属鉴定,在2份样品中检测出了蘑菇毒素,在1份拟灰花纹鹅膏(*Amanita fuligineoides*)干粉提取物中检出*α*-鹅膏毒肽、*β*-鹅膏毒肽和二羟鬼笔毒肽,含量分别为607、377、69.0 mg/kg;在1份口蘑科(Tricholomataceae)蘑菇中检出脱磷裸盖菇素,含量为12.6 mg/kg。

## Abstract (en)

The discovery and identification of mushroom toxins has long been an important area in the fields of toxicology and food safety. Mushrooms are widely favored for their culinary and medicinal value; however, the presence of potentially lethal toxins in some species poses a substantial challenge in ensuring their safe consumption. Therefore, the development of a robust and sensitive analytical method is necessary for accurately identifying the risks associated with mushroom consumption. The study of mushroom toxins, which are characterized by their diversity and substantial variations in chemical structures, present a considerable challenge for achieving precise and high-throughput analysis.

To address this issue, the present study employed a robust approach combining a solid-phase extraction (SPE) purification technique with high performance liquid chromatography-tandem mass spectrometry (HPLC-MS/MS) to establish an analytical method for the detection and quantification of five amatoxins and two tryptamines (psilocybin and bufotenine) present in some mushrooms. Several optimization procedures were undertaken, including optimizing the chromatographic conditions, mass spectrometric parameters, and sample extraction and purification. The procedure involved the extraction of dry mushroom powder with methanol containing 0.3% formic acid, followed by purification using a strong cation exchange cartridge (SCX). The analytes were separated on a T3 chromatographic column (100 mm×2.1 mm, 1.8 μm) using mobile phases of acetonitrile and 5 mmol/L ammonium acetate solution containing 0.1% formic acid. The multiple reaction monitoring (MRM) mode was employed for data acquisition. Amatoxins were quantified using matrix-matched standard calibration curves, whereas isotopic internal standards were used to quantify tryptamine. The results showed that all seven toxins exhibited good linearities (*r*^2^>0.99) within the optimized concentration range. The limits of detection (LODs) for bufotenine, psilocybin, and amatoxins were determined as 2.0, 5.0, and 10 μg/kg, respectively, while the limits of quantification (LOQs) were determined as 5.0, 10, and 20 μg/kg, respectively. The LOD and LOQ values further underscore the ability of the method to detect minute quantities of toxins, making it particularly well suited for screening food samples for potential contamination. Using dried shiitake mushroom powder as the matrix, the recoveries of the two tryptamines ranged from 80.6% to 117%, with relative standard deviations (RSDs) ranging from 1.73% to 5.98%, while the recoveries of amatoxins ranged from 71.8% to 115%, with RSDs varying from 2.14% to 9.92% at the three concentration levels. The consistent and satisfactory recoveries of amatoxins and tryptamines demonstrated the ability of this method to accurately quantify the target analytes even in a complex matrix. Comparison with the results of supplementary test method recognized by State Administration for Market Regulation for food (BJS 202008) demonstrated comparable results, indicating no significant differences (*p*>0.05) in amatoxin contents. The newly developed method is rapid, accurate, precise, meets the required standards, and is suitable for the detection of seven toxins in wild mushrooms. As part of the application of this method, a comprehensive investigation of the distribution of toxins in wild mushrooms from Fujian Province was undertaken. In this study, 59 wild mushroom samples from nine cities were collected in the Fujian province. Species identification was conducted using rDNA-internal transcribed space (rDNA-ITS) molecular barcode technology, which revealed the presence of toxins in the two samples. Notably, one specimen named *Amanita fuligineoides* contained *α*-amanitin, *β*-amanitin, and phalloidin in quantities of 607, 377, and 69.0 mg/kg, respectively. Additionally, another sample, identified as Tricholomataceae, had a psilocybin concentration of 12.6 mg/kg.

世界上有14000多种大型野生真菌^[1]^,其中5000余种为有毒蘑菇;我国野生真菌有3800多种,其中480多种为有毒蘑菇^[2]^。蘑菇富含多糖且脂肪含量低,是世界卫生组织(WHO)和联合国粮食及农业组织(FAO)推荐的“超级食品”^[3]^,但可食用蘑菇和有毒蘑菇外形上极易混淆,我国野生蘑菇中毒事件频发,多年来毒蘑菇一直是引起我国食源性中毒死亡的主要致病因子^[4]^。根据结构和中毒机理,蘑菇毒素可分为鹅膏肽类、毒蝇碱、色胺类、异恶唑衍生物、鹿花菌素、鬼伞素及奥来毒素7类^[5]^,已确定的毒性较强及比较常见的蘑菇毒素主要有鹅膏肽类及色胺类等。其中鹅膏肽类包括鹅膏毒肽(amatoxins)和鬼笔毒肽(phallotoxins)等,色胺类包含脱磷裸盖菇素(psilocin)和蟾蜍色胺(bufotenine)等,都是常见的神经精神型蘑菇毒素^[6⇓-8]^,脱磷裸盖菇素于1958年从墨西哥裸盖菇(*Psilocybe mexicana*)^[9]^中分离得到,分布在花斑褶伞菇(*Panaeolus retirugis Fr. Gill*.)等150多种蘑菇中,是国家管制的精神类药品^[10]^和致幻剂^[11,12]^,易被用作毒品,食用后会出现视幻觉、狂笑、过度兴奋等^[13]^。蟾蜍色胺是蛤蟆菌(*Amanita muscaria*)中的主要致幻成分,能引起色彩幻觉,在美国、英国和澳大利亚被归为违禁物质^[14]^,在人体内不易被分解,即使排泄后仍有致幻作用。

关于鹅膏毒肽和鬼笔毒肽的研究已有不少报道,检测方法也较为成熟^[15⇓⇓⇓⇓⇓⇓-22]^,国家市场监管总局发布了食品补充检验方法BJS 202008^[23]^,该法采用亲水亲脂固相萃取柱(HLB)结合LC-MS/MS测定蘑菇中6种鹅膏肽类毒素,具体为*α*-鹅膏毒肽(*α*-AMA)、*β*-鹅膏毒肽(*β*-AMA)、*γ*-鹅膏毒肽(*γ*-AMA)、羧基二羟鬼笔毒肽(PCD)、羧基三羟鬼笔毒肽(PSC)、二羟鬼笔毒肽(PHD), 6种鹅膏肽类的检出限(LOD)、定量限(LOQ)分别为10、30 μg/kg。关于色胺类毒素检测的研究报道较少,Martin等^[14]^采用混合型阳离子交换柱(MCX)结合LC-MS/MS建立了生物样品(血清、血浆和尿液)中脱磷裸盖菇素、蟾蜍色胺的测定方法,LOD为0.1~0.2 μg/L; Bambauer等^[12]^建立了聚合型强阳离子交换柱(Strata X-Drug B)结合液相色谱-高分辨质谱法(LC-HRMS)检测尿液中鹅膏肽类(*α*-鹅膏毒肽、*β*-鹅膏毒肽)、色胺类毒素(脱磷裸盖菇素、蟾蜍色胺)和神经精神型蘑菇毒素(鹅膏蕈氨酸、毒蝇母、毒蝇碱)的方法,鹅膏毒肽、色胺类的LOD分别为1、5 μg/L。徐小民等^[24]^建立了蘑菇中5种鹅膏肽类和4种神经精神型蘑菇毒素(鹅膏蕈氨酸、毒蝇母、毒蝇碱和裸盖菇素)的测定方法,样品经提取、稀释后用LC-MS/MS测定,LOD较高,为6~15 mg/kg,该方法适合野生菌中蘑菇毒素的检测。

色胺类蘑菇毒素在酸性条件下容易形成阳离子化合物,鹅膏肽类毒素易被反相保留,故主要选取具有阳离子交换和非极性双重作用的固相萃取柱作为净化填料,本研究通过强阳离子固相萃取净化技术结合HPLC-MS/MS,建立了同时测定蘑菇中5种鹅膏肽类和2种色胺类毒素的检测技术,获得了更低的检出限,这将为蘑菇中毒事件的快速诊断和及时治疗提供技术支持。

## 1 实验部分

### 1.1 仪器、试剂与材料

LCMS-8060 NX液相色谱-串联质谱仪(日本岛津公司);冷冻高速离心机(3H24R1,长沙湘智离心机仪器有限公司);漩涡混合器(美国Scientific Industries公司); KQ-500DE型和KQ3200DE型超声波清洗器(昆山市超声仪器公司); EFAA-DC24氮吹仪(上海安谱公司); RE801旋转蒸发仪(日本Yamato公司)。SCX SPE柱(强阳离子交换型, 60 mg/3 mL,美国Agilent公司)。

乙酸铵和甲酸(FA)(色谱纯,CNW公司)、甲酸(优级纯,德国DUKSAN公司)、氨水(优级纯,国药集团,纯度25%~28%)、乙腈(色谱纯,美国ThermoFisher公司)、甲醇(色谱纯,德国Merck公司)、抗坏血酸(CP,天津光复精细化工研究所,纯度≥99.7%)。*α*-鹅膏毒肽、*β*-鹅膏毒肽、*γ*-鹅膏毒肽、羧基二羟鬼笔毒肽和二羟鬼笔毒肽,均来自美国ENZO公司,纯度均≥90%;蟾蜍色胺(天津阿尔塔公司)和脱磷裸盖菇素(加拿大TRC公司),纯度均≥95%。同位素内标标准储备溶液脱磷裸盖菇素-D_10_(100.0 mg/L)、蟾蜍色胺-D_4_(100.2 mg/L)均购自加拿大TRC公司;香菇干粉由购自当地超市的鲜香菇样品烘干后粉碎而得。

### 1.2 标准溶液的配制

毒素混合标准使用液:称取7种蘑菇毒素标准品粉末,分别用甲醇溶解后定容于10 mL容量瓶中,得到质量浓度均为100 mg/L的标准储备液;准确移取1.00 mL 7种标准储备液,用甲醇定容,配制成10.0 mg/L混合标准中间溶液;再准确移取1.00 mL混合标准中间溶液,用甲醇稀释、定容,配制成1.00 mg/L的混合标准使用液。

内标混合标准使用液:取适量2种内标标准储备溶液,用甲醇配制成1.00 mg/L。

### 1.3 样品前处理

称取0.2 g(精确至1 mg)蘑菇干粉于15 mL离心管中,准确加入10 μL 1.00 mg/L内标混合标准使用液,再加入5 mL 0.3%甲酸(优级纯)甲醇溶液,涡旋混匀,在30 ℃下超声提取15 min,以9000 r/min的转速离心10 min,再重复提取一次,合并提取液,添加10%(质量分数,下同)抗坏血酸溶液50 μL后,提取液于40 ℃下减压蒸馏至近干,用2 mL 0.3%甲酸水溶液(含0.05%抗坏血酸)洗涤鸡心瓶内壁。将溶液转入用3 mL乙腈、0.3%甲酸水溶液活化平衡好的SCX柱中,分别用1 mL 0.3%甲酸水溶液、1 mL 0.1%甲酸水溶液淋洗,然后用2 mL氨水-甲醇(5∶95, v/v)洗脱,于40 ℃下氮吹至近干;准确加入1.00 mL 0.05%抗坏血酸溶液复溶,混匀后,以12000 r/min转速离心5 min,取上清液测定。

### 1.4 仪器条件

液相色谱条件 HSS T3柱(100 mm×2.1 mm, 1.8 μm,美国Waters公司);柱温:40 ℃;流动相A: 5 mmoL/L乙酸铵水溶液(含0.1%甲酸(色谱纯));流动相B:乙腈;梯度洗脱程序:0~3.0 min, 5%B; 3.0~5.0 min, 5%B~40%B; 5.0~6.0 min, 40%B~95%B; 6.0~7.0 min, 95%B; 7.0~7.5 min, 95%B~5%B; 7.5~10.0 min, 5%B。流速:0.3 mL/min;进样量:10 μL。

质谱条件 电喷雾离子源正离子模式监测(ESI^+^);雾化气、干燥气和加热气分别为氮气、氮气和空气,流量分别为3.0、10和15 L/min;碰撞气:氩气;脱溶剂管、接口和加热块温度分别为250、400和400 ℃; 7种蘑菇毒素及其内标的监测离子对及质谱参数见表1。

**表 1:** 7种蘑菇毒素、同位素内标的质谱参数及保留时间

Compound	Precursor ion (m/z)	Product ion (m/z)	CE/eV	Q1/V	Q3/V	Retention time/min
α-Amanitin (α-AMA)	919.3	259.1^*^	45	26	24	5.60
		901.2	24	22	32	
β-Amanitin (β-AMA)	920.3	259.1^*^	40	26	25	5.59
		902.5	27	26	32	
γ-Amanitin (γ-AMA)	903.3	243.1^*^	42	26	24	5.77
		885.2	28	26	22	
Phallacidin (PCD)	847.3	157.2^*^	45	20	29	5.95
		674.3	35	20	32	
Phalloidin (PHD)	789.3	157.2^*^	55	22	27	5.94
		330.1	45	24	21	
Psilocin	205.1	58.2^*^	16	10	21	5.15
		160.2	19	10	30	
Bufotenine	205.1	58.2^*^	15	10	20	3.22
		160.1	20	23	28	
Psilocin-D_10_	215.2	66.2^*^	17	11	24	5.12
		164.1	22	11	28	
Bufotenine-D_4_	209.2	164.1^*^	19	23	29	3.30
		60.1	15	24	23	

* Quantitative ion. CE: collision energy; Q1: Q1 prepole bias voltage; Q3: Q3 prepole bias voltage.

### 1.5 统计分析

实验数据以“平均值±标准差”表示,采用Excel整理数据,SPSS 25.0进行实验数据分析。各指标数据均进行正态性检验和方差齐性检验,两组间比较采用双侧检验,检验水准*α*设定为0.05,当*p*<0.05表示差异有统计学意义。当数据满足方差齐性,进行*t*检验;数据不满足方差齐性时,进行非参数检验。

## 2 结果与讨论

### 2.1 质谱条件的优化

5种鹅膏肽类毒素均含有羟基、氨基和羰基等易于电离并产生正离子的官能团,因此可用ESI^+^或ESI^-^的一种模式测定。优化过程中,7种毒素在ESI^+^模式下的信号均高于ESI^-^, [M+H]^+^的信号响应均高于[M+Na]^+^。采用MRM模式采集,通过Q3全扫描确认各个毒素的准分子离子[M+H]^+^,各毒素化学结构与二级质谱碎裂途径见图1。

**图1:**
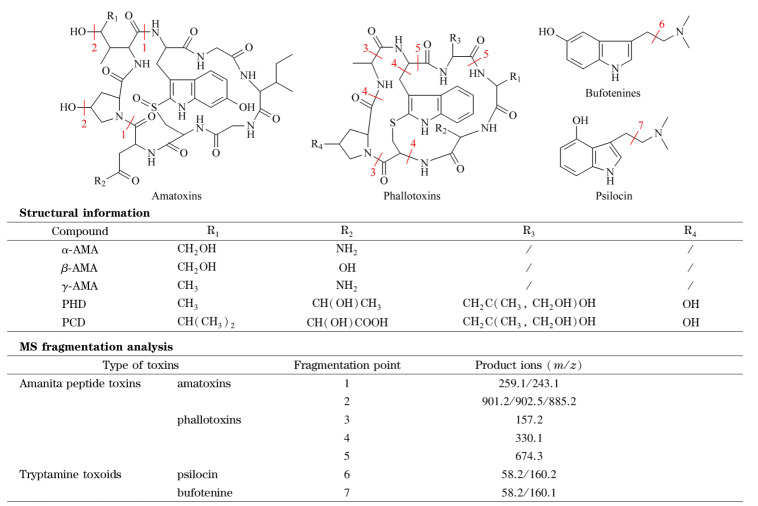
7种蘑菇毒素的结构及质谱碎裂解析

对各个毒素二级碎裂离子进行扫描优化得到最佳碰撞能量及偏差电压,每个毒素得到的离子对信息均能通过碎裂解析找到对应的碎裂部位。比较在优化后的仪器条件下不同基质中毒素的响应,基质干扰等问题,确定了用于定量和定性的产物离子,见表1。

### 2.2 色谱条件优化

在流动相优化过程中,对比了0.1%甲酸水溶液-甲醇、0.1%甲酸水溶液-乙腈,发现乙腈作为有机相时毒素信号响应及峰形普遍比用甲醇好。BJS 202008^[23]^采用甲酸铵溶液,其他文献多采用乙酸铵溶液^[25]^或甲酸水溶液^[24]^作为水相对5种鹅膏肽类毒素进行检测。本研究以乙腈为有机相,比较了水相为水、0.1%甲酸水溶液、5 mmoL/L甲酸铵溶液、5 mmoL/L乙酸铵溶液(含0.1%甲酸)时的效果,发现以5 mmoL/L乙酸铵(含0.1%甲酸)为流动相水相时,所有毒素的信号响应和峰形的综合效果最好,故选择5 mmoL/L乙酸铵(含0.1%甲酸)-乙腈为最终流动相。

蟾蜍色胺、脱磷裸盖菇素互为同分异构体,需要在色谱上分离开,为此比较了在相同条件下Shim-pack Scepter C_18_(100 mm×2.1 mm, 1.9 μm,日本岛津公司)、HSS T3、Shim-pack Velox HILIC(100 mm×2.1 mm, 2.7 μm,日本岛津公司)3种色谱柱的分离效果(见图2)。结果表明,HILIC柱的分离效果较差,且峰形不理想,T3柱和C_18_柱较为理想,分离效果相当,T3柱对色胺类(脱磷裸盖菇素和蟾蜍色胺)的保留效果略好,综上,选择T3柱。

**图2:**
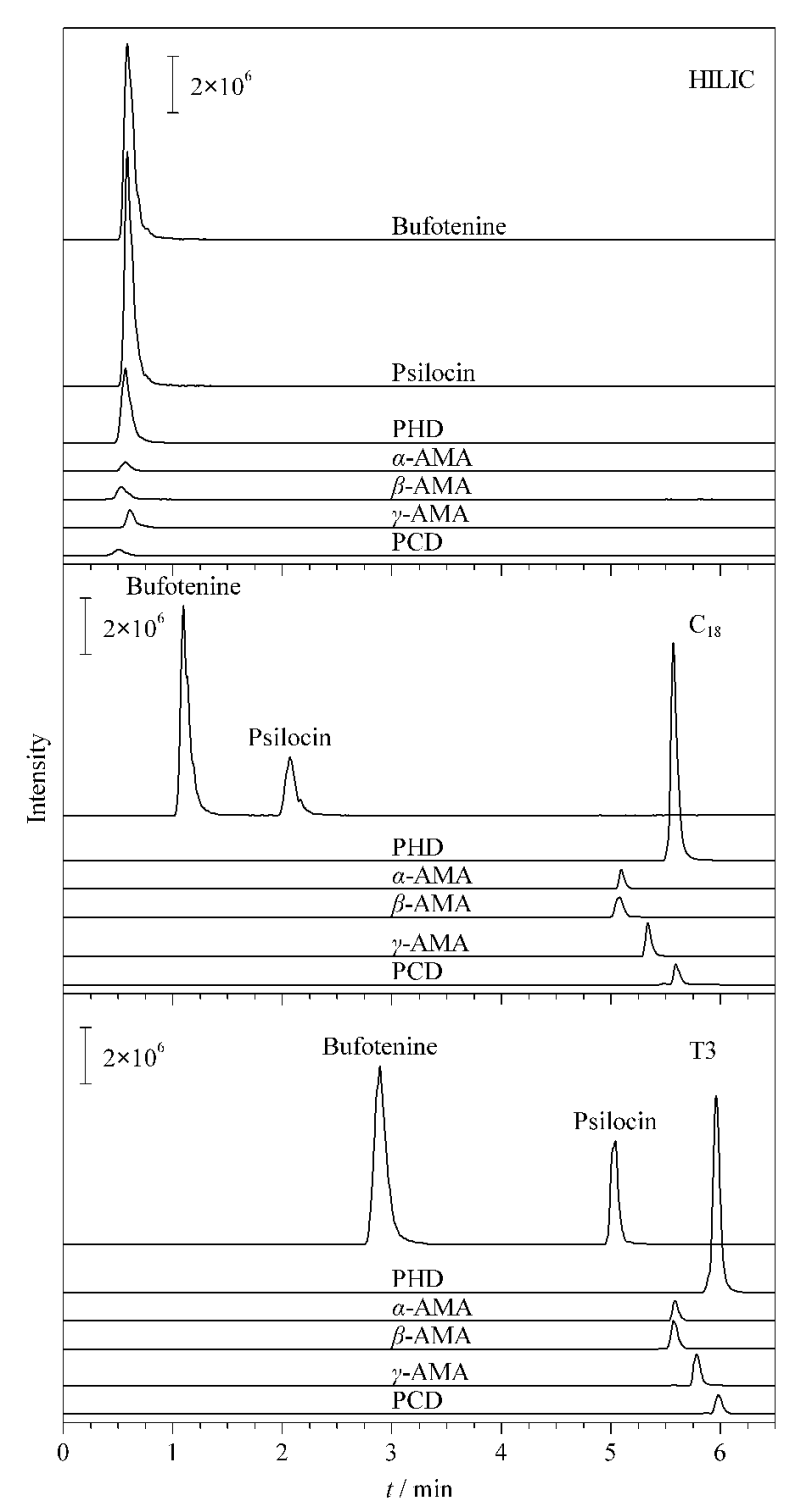
3种色谱柱分离7种蘑菇毒素的MRM色谱图的比较

### 2.3 样品前处理条件的优化

#### 2.3.1 提取溶剂、pH及温度的选择

脱磷裸盖菇素不稳定,在光和空气中会快速地分解,本研究在样品前处理过程中,通过加入抗坏血酸来提高脱磷裸盖菇素的稳定性^[14]^。鹅膏肽类毒素均为多肽类化合物,BJS 202008^[23]^采用甲醇提取,其他文献则多采用乙腈^[26]^、水^[18]^提取;脱磷裸盖菇素和蟾蜍色胺均属于小分子极性化合物,易溶于水。为实现蘑菇中7种蘑菇毒素的提取,对比了水、甲醇、乙腈、30%甲醇水溶液、30%乙腈水溶液的提取效果,结果见图3a。

**图3:**
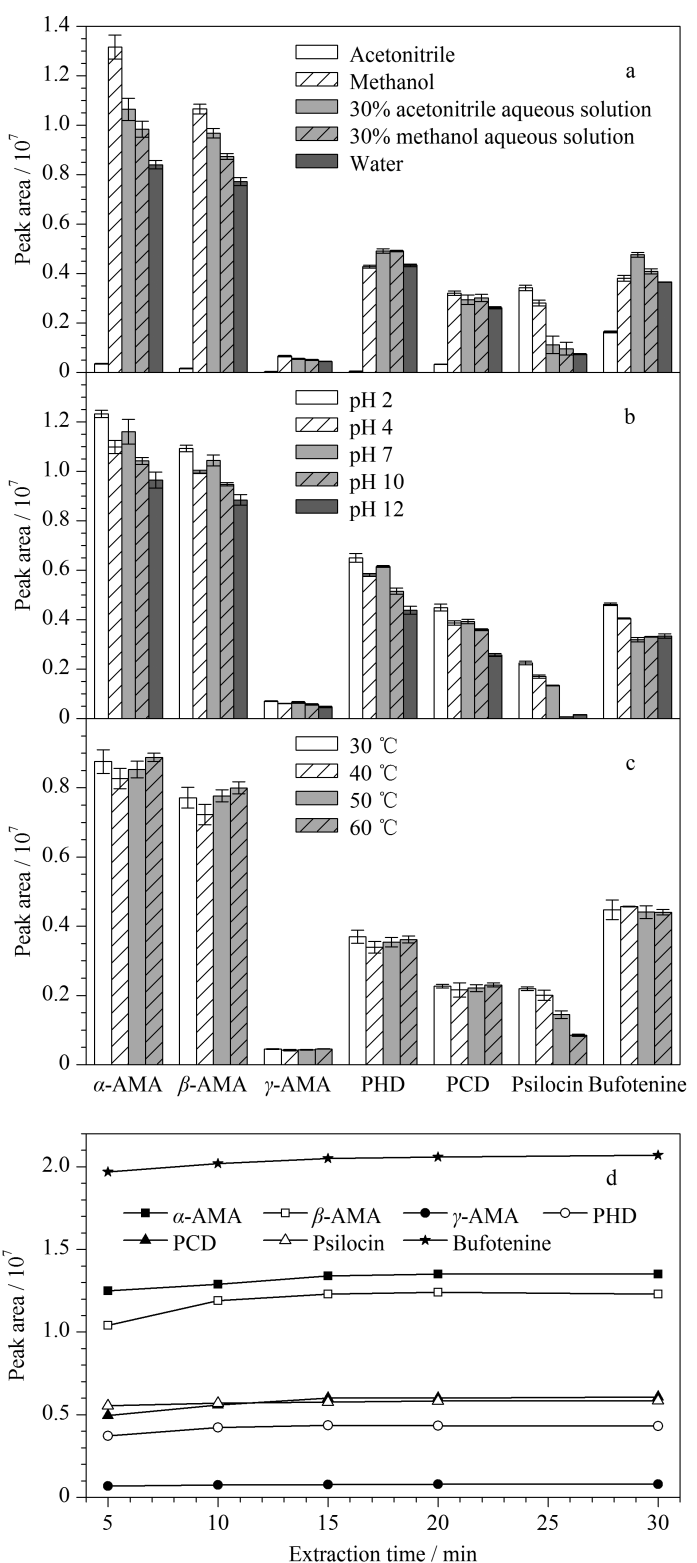
提取条件对蘑菇样品中蘑菇毒素提取效率的影响(*n*=3)

甲醇对PHD的提取效果略低于30%甲醇水和30%乙腈水溶液,对其他鹅膏肽类毒素的提取效果均最佳,提取率略大于30%乙腈水溶液,鹅膏肽类毒素属于相对分子质量较大的极性化合物,相对于甲醇和水,极性更小的乙腈提取率明显更低;对脱磷裸盖菇素及蟾蜍色胺,乙腈和30%乙腈水溶液的提取效果最佳。综上,选择甲醇作为最佳的提取溶剂。

不同的鹅膏肽类毒素具有不同的水溶性,因此推测溶液pH对其提取效果有一定影响^[17]^。本研究对比了在不同pH (2、4、7、10、12)下甲醇溶液对目标物的提取效果,以甲酸溶液及氨水调节提取液的pH值(0.3%甲酸甲醇对应pH 2, 0.1%甲酸甲醇对应pH 4, 0.01%甲酸甲醇对应pH 7,氨水-甲醇(1∶200, v/v)对应pH 10,氨水-甲醇(3∶200, v/v)对应pH 12),结果见图3b。随着pH上升,所有毒素的提取效果均有不同程度的下降,pH为2,即0.3%甲酸甲醇作为提取溶液时所有毒素提取效果最佳。溶剂pH对脱磷裸盖菇素提取的影响较大,对*γ*-鹅膏毒肽提取的影响较小。

鹅膏肽类毒素性质稳定,耐高温、耐干燥和耐酸碱,普通烹调加工无法降低其毒性^[16]^。考察了在30~60 ℃水浴下提取7种毒素时其提取效果的差异(见图3c),脱磷裸盖菇素在30~60 ℃范围内,随着温度升高,其峰面积呈下降趋势(*p*<0.05),这说明脱磷裸盖菇素不耐热,其余6种毒素在不同温度下提取时的峰面积基本一致,无显著性差异(*p*>0.05);综合考虑,确定提取温度为30 ℃。另外试验了30 ℃下提取不同时间(5~30 min)对蘑菇毒素含量的影响(见图3d),大多数毒素在15 min后峰面积基本不变,表明提取达到动态平衡。

#### 2.3.2 固相萃取填料的比较

蘑菇中含有较多的多糖、氨基酸、多肽及有机酸等,基质复杂,BJS 202008^[23]^以及其他文献^[16,25]^多采用亲水亲脂平衡机制的HLB柱作为鹅膏肽类毒素测定的净化柱,对于脱磷裸盖菇素和蟾蜍色胺的测定,多采用离子交换型净化柱^[14]^。因此本研究考察了4种不同类型固相萃取柱的效果,前期试验发现,在提取液上样后,脱磷裸盖菇素未与HLB填料作用,随溶液直接流出;考虑脱磷裸盖菇素和蟾蜍色胺在酸性条件下可形成阳离子,故比较了具有阳离子交换和反相保留双重作用的3种填料WCX(弱阳离子交换型)、MCX、SCX,并用加标水平为50 μg/kg的香菇干粉为基质进行试验,发现SCX对7种毒素净化的综合效果最佳,结果见图4。

**图4:**
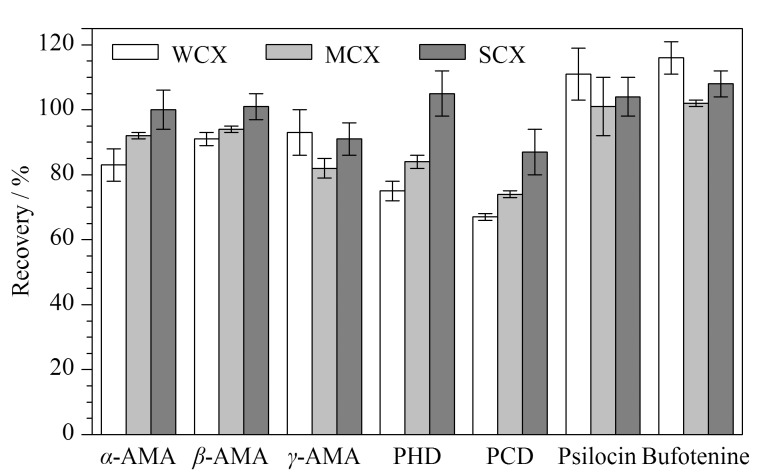
采用3种SPE填料净化时7种蘑菇毒素的回收率(*n*=3)

#### 2.3.3 淋洗和洗脱条件的优化

对SCX净化柱的淋洗液进一步优化,比较方案1(依次用0.3%FA、0.1%FA各1 mL淋洗)和方案2(依次用0.3%FA、5%甲醇(含0.1%FA)各1 mL淋洗)的效果。方案1中7种毒素的回收率范围为86%~126%,方案2的回收率为83%~130%,可见两种淋洗条件下7种毒素的回收率无明显差异。排除掉脱磷裸盖菇素和蟾蜍色胺的内标校正作用后,脱磷裸盖菇素受淋洗溶剂的影响大,采用方案2条件淋洗时,其峰面积仅为方案1的39%(结果见图5),故选择方案1更适合。

**图5:**
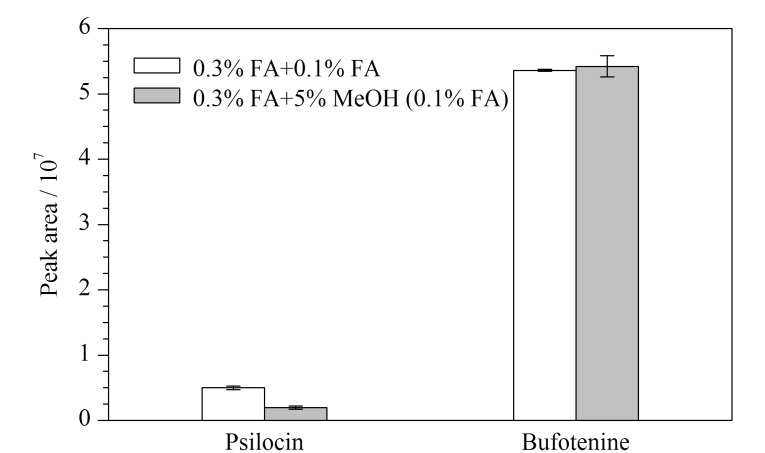
两种淋洗方式下脱磷裸盖菇素和蟾蜍色胺的峰面积(*n*=3)

色胺类毒素用SPE净化时,文献多采用氨水-甲醇洗脱^[12,14]^,本文以氨水-甲醇(5∶95, v/v)洗脱,并对比洗脱体积(1~4 mL)的效果,结果见图6。洗脱液为2 mL时,5种鹅膏肽类毒素的回收率为71%~105%,色胺类毒素的回收率为78%~111%,效果最佳。洗脱液为4 mL时,5种鹅膏肽类毒素的回收率降至56%~65%,这可能是由于随着洗脱体积的增加,导致样品中更多杂质被洗脱出来,基质抑制效应增加,用外标法计算时其回收率较低;2种色胺类毒素的回收率为73%~113%,回收率无明显差异,这可能因为内标法校正后减弱了基质干扰作用。

**图 6:**
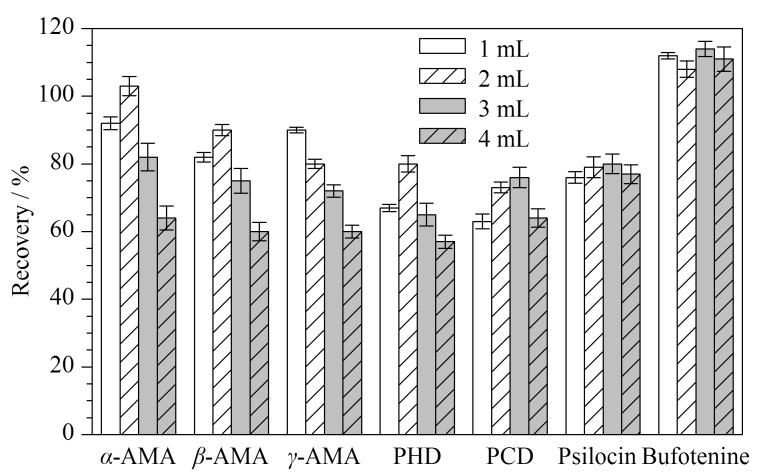
洗脱体积对7种蘑菇毒素回收率的影响(*n*=3)

### 2.4 方法学考察

#### 2.4.1 线性范围、检出限及定量限

按 1.3 节处理得到空白基质溶液,取 7 种蘑菇毒素混合标准使用液适量和10 μL 1.00 mg/L 内标混合标准使用液用空白基质溶液稀释,配制成基质匹配混合标准溶液。鹅膏毒肽、鬼笔毒肽的定量采用基质匹配外标法,以毒素的质量浓度(*x*,μg/L)和峰面积(*y*)拟合线性回归方程;脱磷裸盖菇素和蟾蜍色胺的定量采用同位素内标法,以毒素的质量浓度(*x*,μg/L)与峰面积比值(*Y*)拟合线性回归方程,结果见表2。鹅膏毒肽、鬼笔毒肽(0.5~500 μg/L)、脱磷裸盖菇素(0.1~60 μg/L)、蟾蜍色胺(0.1~60 μg/L)在线性范围内相关系数(*r*^2^)均≥0.997,满足测定要求。取空白香菇干粉进行加标回收试验,以离子对的信噪比≥3和≥10时的含量分别作为LOD和LOQ, 7种毒素的结果见表2。蟾蜍色胺、脱磷裸盖菇素、鹅膏肽类毒素的LOD分别为2.0、5.0、10 μg/kg, LOQ分别为5.0、10和20 μg/kg。BJS 202008^[23]^中称样量(干粉)为0.4 g时,5种鹅膏肽类的LOD均为10 μg/kg, LOQ均为30 μg/kg。本研究的称样量为0.2 g,5种鹅膏肽类的检出限与食品补充检验方法相当,定量限低于该方法,证明本研究方法的灵敏度较高,需要的样品量更少。此外,新建立的方法还能额外检测野生菌中的色胺类毒素(脱磷裸盖菇素、蟾蜍色胺)。

**表 2:** 蘑菇干粉中7种毒素的线性关系、检出限和定量限

Compound	Regression equation	r^2^	Linear range/ (μg/L)	LOD/ (μg/kg)	LOQ/ (μg/kg)
α-AMA	y=8262.2x-2551.9	0.998	0.5-500	10	20
β-AMA	y=9296.5x-234.71	0.998	0.5-500	10	20
γ-AMA	y=15794x-9035.9	0.997	0.5-500	10	20
PCD	y=7448.8x+10336	0.997	0.5-500	10	20
PHD	y=33411x-99875	0.998	0.5-500	10	20
Psilocin	Y=0.081x-0.0189	0.999	0.1-60	5.0	10
Bufotenine	Y=0.2203x+0.0456	0.998	0.1-60	2.0	5.0

*y*: peak areas of amatoxins; *Y*: peak area ratios of two tryptamines to internal standards; *x*: mass concentrations of the seven mushroom toxins, μg/L.

#### 2.4.2 准确度与精密度

以不含目标毒素的香菇干粉进行加标回收率试验,加标水平为5~100 μg/kg时,7种毒素的平均回收率为71.8%~117%, RSD为1.73%~9.92%(*n*=6)(见表3)。根据GB/T 27417-2017^[27]^,当目标物的含量≤0.1 mg/kg时,回收率允许范围为60%~120%,且重复测定的RSD≤15%,这说明本方法的重复性和准确度均理想,满足实际样品的分析要求。

**表 3:** 3个加标水平下香菇干粉中7种蘑菇毒素的加标回收率及其RSD(*n*=6)

Compound	Low			Medium			High	
	Recovery/%	RSD/%		Recovery/%	RSD/%		Recovery/%	RSD/%
α-AMA	88.5	5.83		76.5	7.52		109	4.57
β-AMA	81.6	7.86		72.8	5.62		97.8	3.86
γ-AMA	78.0	5.24		75.0	6.33		115	4.15
PCD	93.4	5.53		75.3	9.92		101	4.03
PHD	89.5	6.26		71.8	3.59		113	2.14
Psilocin	80.6	1.73		89.1	2.83		105	4.05
Bufotenine	117	5.98		113	3.34		109	1.89

Low, medium, high levels: 20, 50, 100 μg/kg for amanita peptide toxins; 10, 20, 100 μg/kg for psilocin; 5, 10, 50 μg/kg for bufotenine.

### 2.5 野生蘑菇中7种蘑菇毒素分布的调查研究

2021年福建省首次开展了野生蘑菇的风险监测,由全省9个设区市采集59份野生蘑菇,本实验室采用核糖体DNA内转录间隔区(rDNA-ITS)分子条形码技术^[28]^进行了种属鉴定(经中国科学院昆明植物研究所杨祝良教授团队核实),并采用本方法测定了野生蘑菇样品中的7种蘑菇毒素。结果显示,在2份样品中检出了蘑菇毒素,编号为LY21615的样品采自龙岩市,经鉴别为拟灰花纹鹅膏(*A*. *fuligineoides*),从中检出*α*-AMA、*β*-AMA、PHD鹅膏肽类毒素,其含量分别为607、377、69.0 mg/kg;编号为PT21097的样品采自莆田市,经鉴别为口蘑科(Tricholomataceae)蘑菇,从中检出脱磷裸盖菇素,含量为12.6 mg/kg,未检出其他6种蘑菇毒素。这两个野外环境下的蘑菇样品分别见图7a和图7c,其干粉提取物的MRM色谱图(各稀释10000、1000倍后测定)分别见图7b和图7d。采用BJS 202008^[23]^方法和本方法对拟灰花纹鹅膏干粉中鹅膏肽类毒素含量进行对比(结果见图8),二者测定的鹅膏肽毒素含量无统计学差异(*p*>0.05),再次证明了本方法的可靠性。

**图7:**
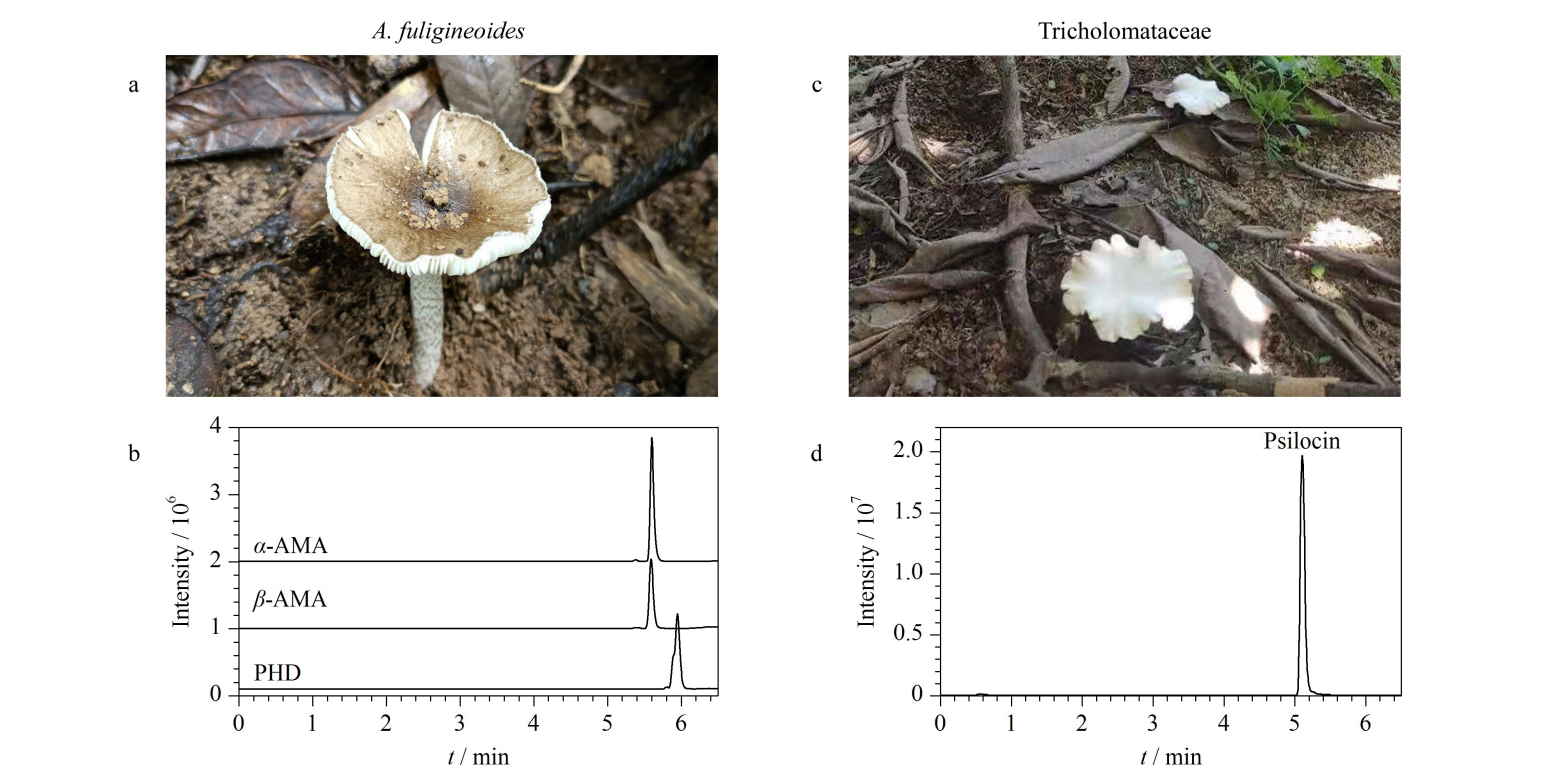
阳性野生蘑菇及其含有的蘑菇毒素的MRM色谱图

**图8:**
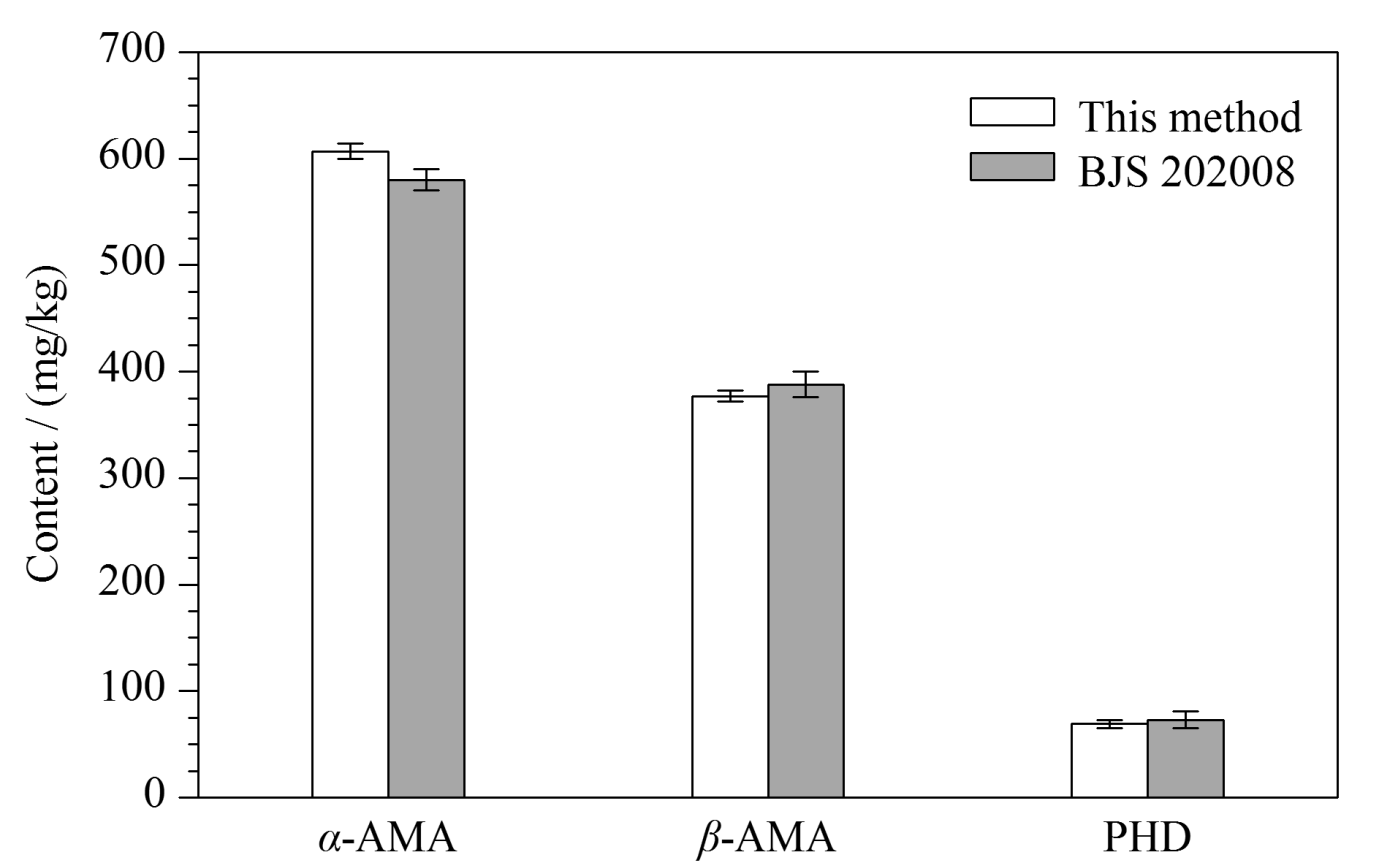
本方法与BJS 202008法检测拟灰花纹鹅膏干粉中鹅膏毒素含量的比较(*n*=3)

## 3 结论

采用强阳离子交换SPE净化柱减少了蘑菇基质对毒素质谱测定的干扰,通过优化前处理条件,建立了蘑菇中5种鹅膏肽类和2种色胺类毒素的LC-MS/MS检测技术。经方法验证并与已有标准方法对比,结果表明所建立方法结果可靠,重复性好,灵敏度较高,可对蘑菇样品中5种鹅膏肽类和2种色胺类蘑菇毒素进行简便、快速、准确的分析。
